# Comparative analysis of convolutional neural networks and transformer architectures for breast cancer histopathological image classification

**DOI:** 10.3389/fmed.2025.1606336

**Published:** 2025-06-17

**Authors:** Bo Yuan, Yudie Hu, Yan Liang, Yutong Zhu, Lingyu Zhang, Shimin Cai, Rui Peng, Xianbin Wang, Zheng Yang, Jinhui Hu

**Affiliations:** The First Hospital of Hunan University of Chinese Medicine, Hunan University of Chinese Medicine, Changsha, China

**Keywords:** breast cancer, deep learning, pathological tissue section, artificial intelligence, foundation model

## Abstract

**Background:**

Breast cancer remains the most prevalent malignancy in women globally, representing 11.7% of all new cancer cases (2.3 million annually) and causing approximately 685,000 deaths in 2020 (GLOBOCAN 2020). This multifactorial disease, influenced by genetic, hormonal and lifestyle factors, often presents with nonspecific early symptoms that delay detection. While mammography, ultrasound and MRI serve as primary screening modalities, histopathological examination remains the diagnostic gold standard—though subject to interpretation variability. Recent advances in deep learning demonstrate promising potential to improve diagnostic accuracy, reduce false positives/negatives, and alleviate radiologists’ workload, thereby enhancing clinical decision-making in breast cancer management.

**Methods:**

This study trains and evaluates 14 deep learning models, including AlexNet, VGG16, InceptionV3, ResNet50, Densenet121, MobileNetV2, ResNeXt, RegNet, EfficientNet_B0, ConvNeXT, ViT, DINOV2, UNI, and GigaPath on the BreakHis v1 dataset. These models encompass both CNN-based and Transformer-based architectures. The study focuses on assessing their performance in breast cancer diagnosis using key evaluation metrics, including accuracy, specificity, recall (sensitivity), F1-score, Cohen’s Kappa coefficient, receiver operating characteristic (ROC) curve, and the area under the ROC curve (AUC).

**Results:**

In the binary classification task, due to its relatively low complexity, most models achieved excellent performance. Among them, CNN-based models such as ResNet50, RegNet, and ConvNeXT, as well as the Transformer-based foundation model UNI, all reached an AUC of 0.999. The best overall performance was achieved by ConvNeXT, which attained an accuracy of 99.2% (95% CI: 98.3%–1), a specificity of 99.6% (95% CI: 99.1%–1), an F1-score of 99.1% (95% CI: 98.0–1%), a Cohen’s Kappa coefficient of 0.983 (95% CI: 0.960–1), and an AUC of 0.999 (95% CI: 0.999–1). In the eight-class classification task, the increased complexity led to more pronounced performance differences among models, with CNN- and Transformer-based architectures performing comparably overall. The best-performing model was the fine-tuned foundation model UNI, which attained an accuracy of 95.5% (95% CI: 94.4–96.6%), a specificity of 95.6% (95% CI: 94.2–96.9%), an F1-score of 95.0% (95% CI: 93.9–96.1%), a Cohen’s Kappa coefficient of 0.939 (95% CI: 0.926–0.952), and an AUC of 0.998 (95% CI: 0.997–0.999). Additionally, using foundation model encoders directly without fine-tuning resulted in generally poor performance on the classification task.

**Conclusion:**

Our findings suggest that deep learning models are highly effective in classifying breast cancer pathology images, particularly in binary tasks where multiple models reach near-perfect performance. Although recent Transformer-based foundation models such as UNI possess strong feature extraction capabilities, their zero-shot performance on this specific task was limited. However, with simple fine-tuning, they quickly achieved excellent results. This indicates that with minimal adaptation, foundation models can be valuable tools in digital pathology, especially in complex multi-class scenarios.

## 1 Introduction

Breast cancer in women has surpassed lung cancer to become the most common cancer worldwide, with both its incidence and mortality rates continuously rising in recent years. It has become a major public health issue that severely threatens women’s health. According to the Global Cancer Statistics (GLOBOCAN 2020), breast cancer has overtaken lung cancer as the most common cancer globally, accounting for 11.7% of all new cancer cases, with an estimated 2.3 million new cases annually. Additionally, breast cancer is one of the leading causes of cancer-related deaths among women worldwide, with approximately 685,000 female deaths attributed to the disease in 2020 (1). In China, breast cancer is the most common malignancy among women, with an estimated 306,000 new cases in 2016 (2). Since the widespread use of mammography, the incidence of breast cancer has steadily increased and continues to rise, exacerbated by the aging population (3).

The etiology of breast cancer involves multiple factors, including genetic, hormonal, lifestyle, and environmental exposures. Among these, mutations in the BRCA1 and BRCA2 genes are considered significant genetic factors in the development of breast cancer (3–5). Furthermore, lifestyle factors such as high-fat diet, obesity, long-term use of estrogen-based medications, smoking, and alcohol consumption are closely associated with an increased risk of breast cancer (6, 7). The early symptoms of breast cancer are often subtle, and many patients are diagnosed only after the disease progresses to advanced stages. Therefore, the establishment of efficient and accurate breast cancer screening methods to improve early diagnosis rates is of crucial significance in reducing mortality and improving patient prognosis.

Currently, the detection methods for breast cancer include X-ray imaging, ultrasound, breast MRI (Magnetic Resonance Imaging), and mammography. However, these imaging techniques provide only preliminary results and cannot definitively determine whether the lesion is malignant. The only reliable method for confirming the presence of cancer is through biopsy, where tissue samples are analyzed pathologically (8). Only after pathological confirmation can the physician establish a clear treatment plan (9). However, when performing biopsy tissue sectioning for pathological examination, it is often challenging to make accurate diagnoses due to factors such as cellular overlap or uneven staining. This process is both time-consuming and labor-intensive, and because of variability in the skill levels of pathologists, different pathologists may arrive at different diagnostic conclusions for the same pathological image.

In recent years, with the explosive growth of medical imaging data, artificial intelligence (AI), particularly deep learning, has shown remarkable potential in the early diagnosis and prognosis prediction of cancer. It first achieved outstanding results in lung cancer screening and diagnosis (10, 11), followed by increasing attention to the application of AI in breast cancer screening and diagnosis (12). Traditional breast imaging diagnosis relies on the subjective judgment of radiologists, and the results are prone to be influenced by factors such as the doctor’s level of experience, fatigue, and image quality, leading to certain rates of misdiagnosis (false positives) and missed diagnoses (false negatives). For example, studies have shown that the false-negative rate of mammography can be as high as 10–30%, meaning that some breast cancer cases may not be detected in the initial screening (13). Moreover, the shortage of imaging specialists, combined with the rapid increase in imaging data, has exacerbated the burden of manual interpretation. As a result, deep learning-based breast cancer imaging analysis technologies hold the potential to significantly improve diagnostic consistency, accuracy, and efficiency.

Deep learning is an important branch of machine learning, particularly proficient in automatically extracting key features from large-scale medical imaging data and performing tasks such as classification, detection, and segmentation. In recent years, multiple studies have demonstrated that deep learning models based on Convolutional Neural Networks (CNN) perform at or even exceed the diagnostic level of radiologists in breast cancer screening (14–16). For example, the AI breast cancer screening system developed by Google Health, when tested on datasets from the UK and the US, reduced the false positive rate by 5.7 and 1.2%, respectively, and decreased the false negative rate by 9.4 and 2.7%, showing its potential in clinical applications (17). In the field of medical image analysis, CNN is one of the most widely applied deep learning architectures. Through multiple layers of convolution, pooling, and fully connected operations, CNNs can automatically learn features from large-scale breast imaging data, extract lesion regions, and perform classification and detection tasks. For instance, CNN variants such as ResNet (18), VGG (19), DenseNet (20), and EfficientNet (21) have been extensively used for breast cancer screening tasks. An AI model based on ResNet50 has been proven to achieve diagnostic performance comparable to senior radiologists on mammography X-ray images. In recent years, Transformer architectures, particularly the Vision Transformer (ViT), have achieved significant breakthroughs in computer vision tasks. Unlike CNN, which primarily relies on local convolution operations, ViT (22) models global information through self-attention mechanisms, enabling it to better capture long-range dependencies in breast images, especially in identifying subtle lesions such as microcalcifications and masses, thus enhancing the ability to detect early-stage breast cancer. Following ViT (22), DINOv2 (23) further enhanced its capabilities by adding registries and training on larger-scale datasets, improving its performance significantly.

In recent years, foundation models have garnered significant attention in the field of artificial intelligence. These models are typically large-scale deep learning architectures pretrained on massive and diverse datasets, exhibiting strong generalization and transfer capabilities across a wide range of downstream tasks. Leveraging self-supervised learning, foundation models are capable of learning rich feature representations from unlabeled data by capturing its underlying structure. Prominent examples include BERT (24) and the GPT (25) series in natural language processing, as well as CLIP (26), DINO (23), and SAM (27) in computer vision. These models have demonstrated outstanding performance in zero-shot, few-shot, and fine-tuning scenarios, becoming a driving force behind the advancement of modern AI. Building on the success of foundation models in natural image and language domains, researchers have recently extended this paradigm to the field of medical imaging driving force behind the advancement of modern AI. Building on the success-resolution images, complex morphological features, and costly annotation processes. This has led to the emergence of pathology foundation models, which are pretrained on large-scale histopathology datasets using self-supervised learning to capture intricate tissue characteristics. Representative models in this field include UNI (28) and Prov-GigaPath (29). UNI (28) is the first general-purpose pathology model trained via self-supervised learning on more than 100,000 diagnostic-grade H&E-stained whole slide images (WSIs), encompassing over 100 million image tiles across 20 major tissue types. UNI outperforms existing state-of-the-art models across 34 representative computational pathology (CPath) tasks and demonstrates strong capabilities in resolution-agnostic classification, few-shot learning, and tumor subtype generalization. On the other hand, Prov-GigaPath (29) is the first foundation model designed for full-slide pathology analysis. It was trained on 1.3 billion 256 × 256 image patches extracted from 171,189 WSIs collected from over 30,000 patients within the Providence healthcare network. Leveraging the novel GigaPath architecture and incorporating LongNet to handle giga-pixel scale context, Prov-GigaPath achieved top performance on 25 out of 26 benchmark tasks, with significant improvements over the second-best method in 18 of them. These models highlight the powerful potential of large-scale, real-world pretraining and long-range context modeling. The advent of such models marks the beginning of the foundation model era in pathology, offering transformative capabilities for intelligent diagnostic support systems and paving the way for enhanced performance and generalization across complex clinical tasks.

**FIGURE 1:**
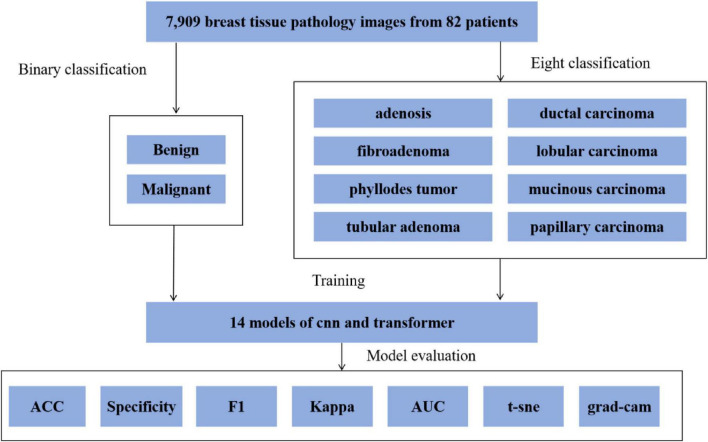
Research framework flowchart.

Building on this foundation, we compared the performance of various deep learning models on this task. The overall pipeline is shown in Figure 1.

## 2 Materials and methods

### 2.1 Data

**FIGURE 2:**
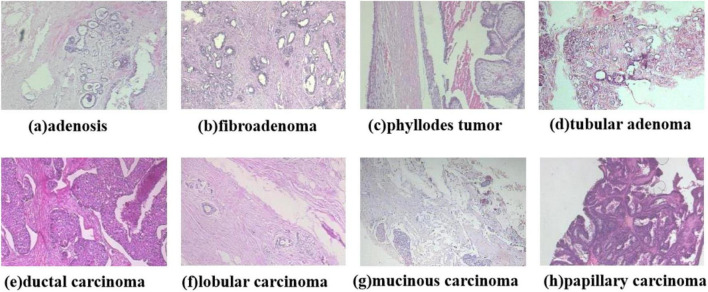
Diagram of the eight types of breast cancer tumor classification.

This study employs the BreaKHis (30) dataset, which was publicly released by Spanhol et al. in 2016 and comprises 7,909 breast histopathological images from 82 patients. All samples were obtained from hematoxylin and eosin (HE)-stained breast tissue biopsy sections and annotated by professional pathologists at the P&D laboratory. The images were acquired using an Olympus BX-50 microscope system in three-channel red-green-blue (RGB) true-color space (24-bit depth, 8 bits per channel) at magnification factors of 40×, 100×, 200×, and 400×. Automatic exposure settings were applied during capture, with manual focusing performed via digitally displayed images on a computer screen. Tumor regions in tissue sections were identified through microscopic visual analysis by anatomical pathologists, with final diagnoses established by senior pathologists incorporating supplementary examinations such as immunohistochemical (IHC) analysis. The BreaKHis dataset provides fine-grained clinical subtype annotations for breast lesions: benign categories include (a) adenosis, (b) fibroadenoma, (c) phyllodes tumor, and (d) tubular adenoma; malignant categories comprise (e) ductal carcinoma, (f) lobular carcinoma, (g) mucinous carcinoma, and (h) papillary carcinoma. The dataset contains 2,480 benign and 5,429 malignant tumor images (700 × 460 pixels, 3-channel RGB format, 8-bit depth per channel, PNG format), with detailed distribution presented in Table 1. The dataset comprises eight classification labels, with representative images shown in Figure 2.

**TABLE 1:** Detailed of the distribution of the dataset.

Magnification factor	Benign	Malignant	Total
40	625	1,370	1,995
100	644	1,437	2,081
200	623	1,390	2,013
400	588	1,232	1,820
Total	2,480	5,429	7,909

### 2.2 Image preprocessing

To begin with, this study implemented data augmentation strategies on the original dataset to enhance training data diversity and improve model generalizability. Conventional augmentation methods typically apply identical transformation operations to all batch data while maintaining the total data volume, dynamically generating differentiated inputs to enrich data variability. In our experiment, we adopted an expansion-based augmentation approach: through randomized operations including random cropping, color space transformation, illumination correction, random rotation, and horizontal flipping, the original dataset was augmented to six times its initial size before being fed into the model for training. During preprocessing, all input images were uniformly resized from the original 700 × 460 pixels to 224 × 224 resolution, except for InceptionV3 (31). Due to its specific architectural requirements, InceptionV3 inputs were adjusted to 299 × 299 pixels (Note: The original mention of 399 × 399 appears to be a typographical error). To ensure training stability and convergence efficiency, we employed Z-score normalization to standardize the image data, transforming pixel value distributions into a normal distribution with zero mean and unit standard deviation, as defined by the following equation:


(1) $$X\prime =\frac{X-\mu }{\sigma }$$

### 2.3 Development of the deep learning system

This study adopted a stratified five-fold cross-validation strategy for model training and validation. Stratified sampling based on class proportions was used to ensure that the class distribution in each validation set remained consistent with that of the entire dataset, thereby effectively preserving data balance and enhancing the reliability of model evaluation. The entire BreaKHis dataset, comprising 7,909 images, was utilized to construct the deep learning system. The experiment systematically evaluated nine classical CNN architectures including AlexNet (32), VGG16 (19), InceptionV3 (31), ResNet50 (18), DenseNet121 (20), MobileNetV2 (33), ResNeXt (34), RegNet (35), and EfficientNet_B0 (21), along with the ConvNeXT (36) architecture combining CNN and Transformer advantages, and pure Transformer-based ViT (22) and DINOv2 (23) models—the latter pretrained through large-scale self-supervised learning on 142 million unlabeled images. All CNN models were initialized with ImageNet (37) pretrained weights, while ViT (22) and DINOv2 (23) utilized parameters pretrained on even larger datasets, as well as the pathology foundation models UNI (28) and GigaPath (29) developed based on the DINO model.

Implemented using the PyTorch framework on NVIDIA 3090 GPUs (24GB VRAM), the experiments set batch size to 128 within VRAM constraints while configuring 20 data loading threads to optimize I/O efficiency. The Adam optimizer was adopted for training, combining adaptive learning rate advantages by updating parameters based on first and second moment estimates of gradients, with initial learning rate set to 0.0001. Cross-entropy loss function with balanced class weighting was applied for optimization (38). At each epoch end, comprehensive model evaluation was conducted by computing validation metrics including loss, accuracy, sensitivity, specificity, Kappa coefficient and AUC, with the highest-AUC model ultimately saved as the optimal result.

### 2.4 Evaluation of the AI system

To comprehensively evaluate the classification performance of our models, we employed multiple metrics including accuracy, sensitivity, specificity, Cohen’s Kappa, F1-score, and AUC (with 95% confidence intervals). All metrics were derived from five-fold cross-validation results, with final values representing the average across all validation folds. Sensitivity and specificity were calculated using a one-vs.-rest strategy, while the 95% confidence intervals for AUC were determined through empirical bootstrap method with 1,000 random samples to ensure robustness. Beyond quantitative metrics, we visually assessed model performance through several techniques. Receiver operating characteristic (ROC) curves (39) demonstrated model performance across different thresholds, where AUC values approaching 1.0 indicated superior classification capability. Comparative analysis of AUC values across multiple models helped identify the optimal architecture. Confusion matrices (40) provided detailed comparisons between true and predicted labels, clearly displaying both correct classifications and misclassifications for each category. This analysis proved particularly valuable for identifying: (1) the actual classes of misclassified samples, and (2) the specific categories these samples were most frequently mistaken for, thereby revealing which lesion types were most prone to confusion during classification. Furthermore, as illustrated in Figures 6, 7, we employed t-distributed stochastic neighbor embedding (t-SNE) (41) to visualize the enhanced separability of features learned by different models in a two-dimensional space. This dimensionality reduction technique provided intuitive insights into how effectively each model distinguished between various pathological subtypes at the feature representation level. To evaluate the zero-shot capability of foundation models, we compared the performance of the UNI and GigaPath encoders when directly applied to the target task versus after fine-tuning.

### 2.5 Interpretability of AI system

To facilitate more intuitive analysis of different models’ focus areas during feature extraction, identify root causes of misclassification, and enhance clinical understanding of deep learning system operations, we employed Gradient-weighted Class Activation Mapping (Grad-CAM) to visually analyze the convolutional neural network models used in our experiments. Grad-CAM generates heatmaps by combining feature maps with gradient information, highlighting the regions in input images that most strongly influence the model’s predictions for specific classes. This visualization approach provides critical insights into the model’s decision-making process by explicitly showing which image regions drive classification decisions. Through this technique, we were able to: (1) verify whether models focused on clinically relevant image regions, (2) identify potential biases in feature attention, and (3) pinpoint anatomical areas that frequently led to misclassifications. The Grad-CAM analysis proved particularly valuable for interpreting model behavior in histopathological contexts, where the spatial distribution of diagnostic features is often crucial for accurate classification.

## 3 Results

### 3.1 Evaluation of deep learning models

The dataset comprising 7,909 images was partitioned into training and validation sets at a 4:1 ratio. The training set contained 6,327 images (1,984 benign and 4,343 malignant), while the validation set consisted of 1,582 images (496 benign and 1,086 malignant). Our study evaluated model performance on two distinct classification tasks: (1) binary classification (benign vs. malignant) and (2) eight-class classification (adenosis, fibroadenoma, phyllodes tumor, tubular adenoma, ductal carcinoma, lobular carcinoma, mucinous carcinoma, and papillary carcinoma). We implemented 12 deep learning architectures for breast tumor classification: nine CNN-based models [AlexNet (32), VGG16 (19), InceptionV3 (31), ResNet50 (18), DenseNet12 (20), MobileNetV2 (33), ResNeXt (34), RegNet (35), and EfficientNet_B0 (21)], one hybrid CNN-Transformer model [ConvNeXT (36)], and two pure Transformer models [ViT (22) and DINOv2 (23)],as well as the pathology foundation models UNI and GigaPath developed based on the DINO model. All models were trained and validated using five-fold cross-validation. Performance metrics included accuracy, sensitivity, specificity, F1-score, weighted Cohen’s Kappa, and AUC with 95% confidence intervals (CI).

**FIGURE 3:**
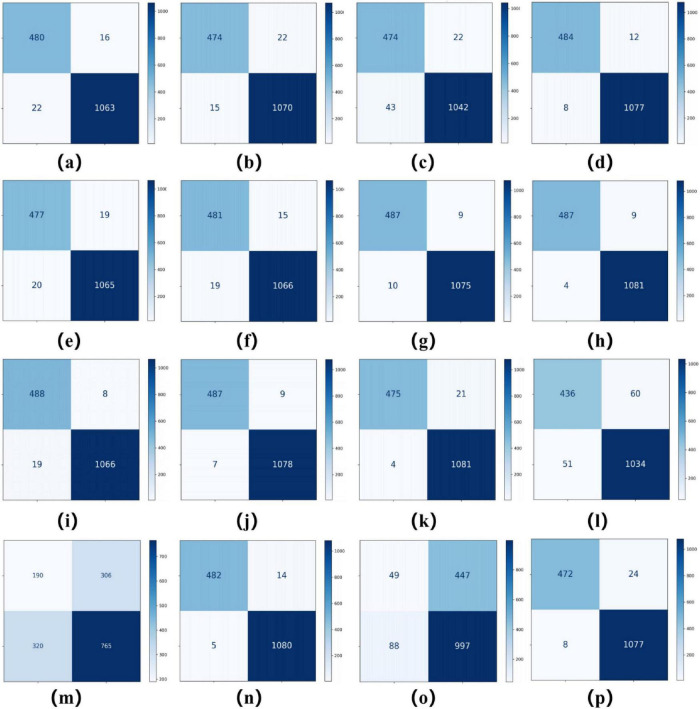
Confusion matrices for 16 models in a binary classification task. Confusion matrices of models **(a–p)**, corresponding to AlexNet, VGG16, InceptionV3, ResNet50, DenseNet121, MobileNetV2, ResNeXt, RegNet, EfficientNet_B0, ConvNeXT, ViT, DINOv2, UNI (zero-shot), UNI (fine-tuned), GigaPath (zero-shot), and GigaPath (fine-tuned).

**FIGURE 4:**
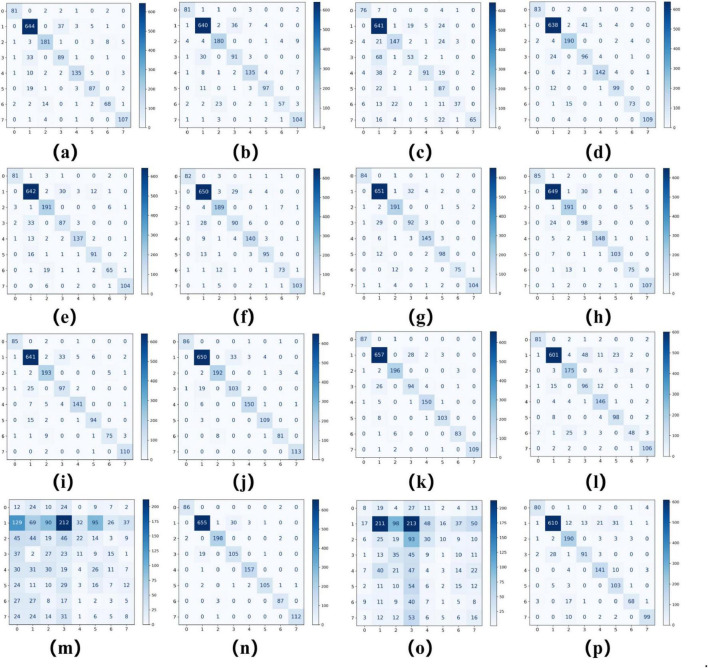
Confusion matrices for 16 models in an eight-class classification task. Confusion matrices of models **(a–p)**, corresponding to AlexNet, VGG16, InceptionV3, ResNet50, DenseNet121, MobileNetV2, ResNeXt, RegNet, EfficientNet_B0, ConvNeXT, ViT, DINOv2, UNI (zero-shot), UNI (fine-tuned), GigaPath (zero-shot), and GigaPath (fine-tuned).

As summarized in Tables 2, 3, ConvNeXT (36) achieved superior performance in binary classification, attaining 99.2% accuracy, 99.6% specificity, 99.1% F1-score, 0.983 Kappa, and 0.999 AUC. For the eight-class task, ResNeXt (34), ViT (22), and ConvNeXT (36) demonstrated optimal performance with 0.996 AUC. Table 4 compares computational characteristics: EfficientNet_B0 (21) showed the fastest training speed, AlexNet (32) had the shortest inference time, and MobileNetV2 (33) contained the fewest parameters, highlighting their respective advantages for different application scenarios. Figures 3, 4 present the confusion matrices for both classification tasks, demonstrating strong overall performance while revealing that fibroadenoma and tubular adenoma were the most frequently confused tumor types in the eight-class classification.

**TABLE 2:** The performance of models for binary classification.

Model	Accuracy (95% CI)	Specificity (95% CI)	F1-score (95% CI)	Kappa (95% CI)	AUC (95% CI)
AlexNet	95.9%	98.4%	95.2%	0.905	0.991
	(93.9%, 97.8%)	(97.6%, 99.1%)	(92.9%, 97.3%)	(0.858, 0.947)	(0.982, 0.997)
Vgg16	98.5%	99.4%	98.3%	0.966	0.997
	(97.1%, 99.5%)	(98.8%, 99.8%)	(96.7%, 99.4%)	(0.933, 0.988)	(0.993, 0.999)
InceptionV3	96.1%	98.0%	95.4%	0.908	0.982
	(94.2%, 97.8%)	(97.0%, 98.9%)	(93.1%, 97.4%)	(0.863, 0.947)	(0.965, 0.995)
ResNet50	98.3%	99.3%	98.0%	0.961	0.999
	(97.1%, 99.2%)	(98.7%, 99.7%)	(96.4%, 99.2%)	(0.929, 0.984)	(0.996, 0.999)
Densenet121	97.1%	98.6%	96.6%	0.932	0.996
	(95.4%, 98.5%)	(97.8%, 99.4%)	(94.5%, 98.2%)	(0.889, 0.965)	(0.992, 0.999)
MobileNetV2	95.9%	97.4%	95.0%	0.900	0.996
	(93.9%, 97.5%)	(96.2%, 98.5%)	(92.6%, 97.1%)	(0.853, 0.942)	(0.992, 0.999)
ResNeXt	97.8%	99.1%	97.4%	0.949	0.997
	(96.3%, 99.0%)	(98.4%, 99.7%)	(95.8%, 98.9%)	(0.915, 0.978)	(0.994, 0.999)
RegNet	98.5%	99.4%	98.3%	0.966	**0.999**
	(97.3%, 99.5%)	(99.0%, 99.9%)	(96.9%, 99.5%)	(0.938, 0.989)	(0.997, 0.999)
EfficientNet_B0	98.7%	99.5%	98.5%	0.971	0.996
	(97.5%, 99.7%)	(98.9%, 99.9%)	(97.1%, 99.7%)	(0.943, 0.994)	(0.992, 0.999)
ConvNeXT	**99.2%**	**99.6%**	**99.1%**	**0.983**	**0.999**
	(98.3%, 1)	(99.1%, 1)	(98.0%, 1)	(0.960, 1)	(0.999, 1)
ViT	98.5%	99.3%	98.3%	0.966	0.996
	(97.3%, 99.5%)	(98.7%, 99.8%)	(96.8%, 99.4%)	(0.937, 0.989)	(0.991, 0.999)
DINOv2	97.8	98.9%	97.4%	0.949	0.998
	(96.1%, 99.0%)	(98.1%, 99.6%)	(95.4%, 98.9%)	(0.909, 0.978)	(0.996, 0.999)
UNI_zero_shot	60.4%	54.4%	54.3%	0.087	0.557
	(57.8%, 62.6%)	(80.6%, 82.7%)	(51.6%, 56.7%)	(0.033, 0.134)	(0.524, 0.585)
UNI_fine-tuning	98.7%	98.3%	98.6%	0.971	**0.999**
	(98.2%, 99.2%)	(99.0%, 99.6%)	(97.9%, 99.1%)	(0.958, 0.982)	(0.998, 1)
GigaPath_Zero-shot	54.4%	52.4%	51.4%	0.043	0.529
	(52.0%, 56.8)	(79.9%, 82.0%)	(48.9%, 53.9%)	(0.041, 0.092)	(0.499, 0.562)
GigaPath_fine-tuning	97.9%	97.2%	97.6%	0.952	0.998
	(97.2%, 98.7%)	(98.4%, 99.2%)	(96.8%, 98.4%)	(0.935, 0.968)	(0.998, 0.999)

Boldface indicates the maximum value in each column.

**TABLE 3:** The performance of models for eight classification.

Model	Accuracy (95% CI)	Specificity (95% CI)	F1-Score (95% CI)	Kappa (95% CI)	AUC (95% CI)
AlexNet	91.8%	97.3%	85.9%	0.844	0.988
	(90.3%, 93.2%)	(96.7%, 97.8%)	(83.8%, 87.8%)	(0.824, 0.863)	(0.985, 0.990)
VGG16	91.4	97.2%	84.7%	0.838	0.987
	(89.9%, 93.0%)	(96.7%, 97.8%)	(82.6%, 86.8%)	(0.817, 0.858)	(0.985, 0.990)
InceptionV3	85.0%	94.0%	69.2%	0.669	0.958
	(82.9%, 87.1%)	(93.2%, 94.7%)	(66.5%, 71.8%)	(0.640, 0.696)	(0.950, 0.964)
ResNet50	92.2%	97.6%	89.5%	0.876	0.991
	(90.8%, 93.6%)	(97.1%, 98.1%)	(87.8%, 91.2%)	(0.858, 0.895)	(0.989, 0.993)
Densenet121	91.9%	97.3%	86.2%	0.848	0.987
	(90.3%, 93.4%)	(96.7%, 97.8%)	(84.1%, 88.1%)	(0.826, 0.869)	(0.984, 0.990)
MobileNetV2	92.5%	97.5%	88.3%	0.869	0.989
	(91.0%, 93.8%)	(97.0%, 98.0%)	(86.5%, 90.0%)	(0.849, 0.886)	(0.986, 0.991)
ResNeXt	**95.9%**	97.4%	95.0%	0.900	0.996
	(93.9%, 97.5%)	(96.2%, 98.5%)	(92.6%, 97.1%)	(0.853, 0.942)	(0.992, 0.999)
RegNet	94.2%	98.1%	91.3%	0.898	0.994
	(92.9%, 95.4%)	(97.7%, 98.5%)	(89.8%, 92.8%)	(0.882, 0.916)	(0.993, 0.996)
EfficientNet_B0	92.7%	97.7%	89.9%	0.881	0.990
	(91.1%, 94.1%)	(97.2%, 98.2%)	(88.2%, 91.6%)	(0.862, 0.899)	(0.986, 0.992)
ConvNeXT	94.2%	**98.3%**	93.6%	0.921	0.996
	(93.3%, 95.8%)	(97.8%, 98.7%)	(92.3%, 94.8%)	(0.904, 0.936)	(0.994, 0.997)
ViT	94.3%	98.1%	93.2%	0.917	0.996
	(93.0%, 95.6%)	(97.7%, 98.5%)	(91.8%, 94.4%)	(0.901, 0.932)	(0.995, 0.997)
DINOv2	90.5%	97.4%	82.2%	0.813	0.987
	(88.7%, 92.0%)	(96.9%, 97.8%)	(80.0%, 84.3%)	(0.792, 0.834)	(0.984, 0.989)
UNI_zero-shot	12.8%	78.3%	8.13%	–0.03	0.438
	(10.7%, 14.9%)	(77.7%, 78.8%)	(6.85%, 9.56%)	(–0.05, –0.02)	(0.421, 0.456)
UNI_fine-tuning	95.5%	95.6%	**95.0%**	**0.939**	**0.998**
	(94.4%, 96.6%)	(94.2%, 96.9%)	(93.9%, 96.1%)	(0.926, 0.952)	(0.997, 0.999)
GigaPath_Zero-shot	27.3%	81.4%	12.2%	0.041	0.542
	(24.4%, 29.9%)	(80.6%, 82.2%)	(10.7%, 13.9)	(0.021, 0.060)	(0.523, 0.559)
GigaPath_fine-tuning	92.2%	97.6%	85.6%	0.838	0.988
	(90.7%, 93.7%)	(97.1%, 98.1%)	(83.5%, 87.4%)	(0.816, 0.858)	(0.984, 0.990)

Boldface indicates the maximum value in each column.

**TABLE 4:** Comparison of different models in terms of training speed, inference speed, number of parameters, and year of release.

Model	Training time (epoch/s)	Infernece time (one/ms)	Number of parameters (M)	Year
AlexNet	36	**0.08**	61	2012
Vgg16	53	0.12	138	2014
InceptionV3	55	1.02	23.8	2015
ResNet50	30	0.39	25.6	2015
Densenet121	35	1.47	7.98	2017
MobileNetV2	39	0.41	**3.4**	2018
RegNet	141	0.93	5.1	2020
EfficientNet_B0	**21**	0.98	5.3	2021
ResNeXt	38	0.76	25	2017
ViT	91	0.43	85.8	2020
ConvNeXT	40	0.77	79	2022
DinoV2	95	0.52	86	2023
UNI	501	0.46	681	2024
GigaPath	810	1.08	1135	2024

Bold font indicates the smallest number of parameters in the “number of parameters” column, while in the other columns, bold font indicates the largest values.

**FIGURE 5:**
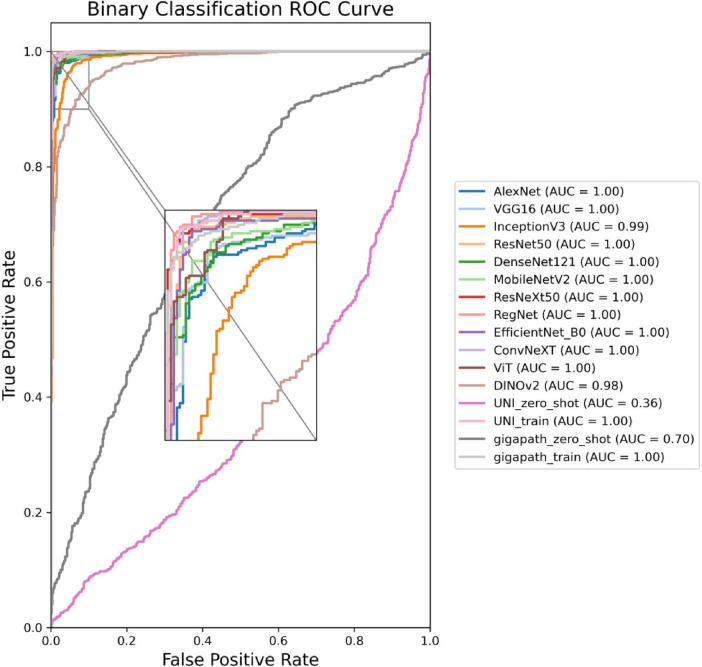
ROC curves for the 16 models in a binary classification task. The model names and their corresponding colors are shown in the bottom right corner of the figure.

**FIGURE 6:**
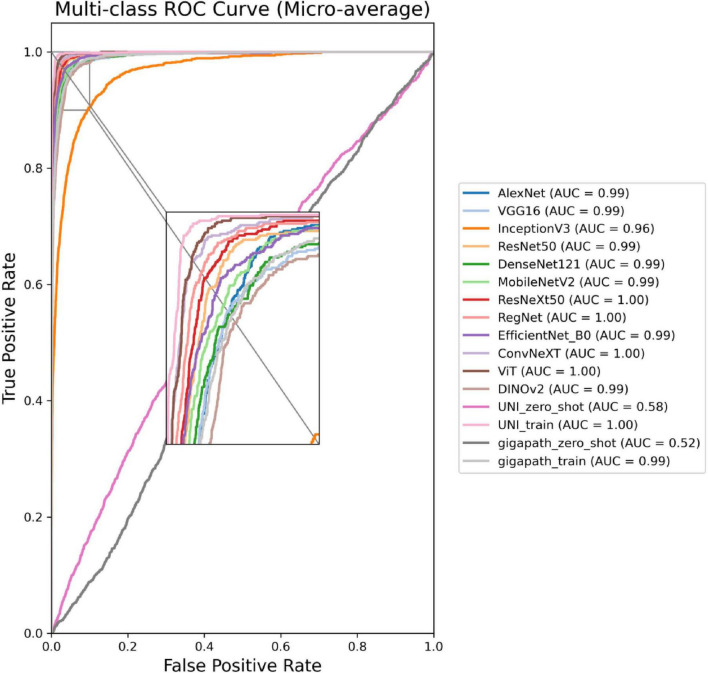
ROC curves for the 16 models in an eight-class classification task. The model names and their corresponding colors are shown in the bottom right corner of the figure.

**FIGURE 7:**
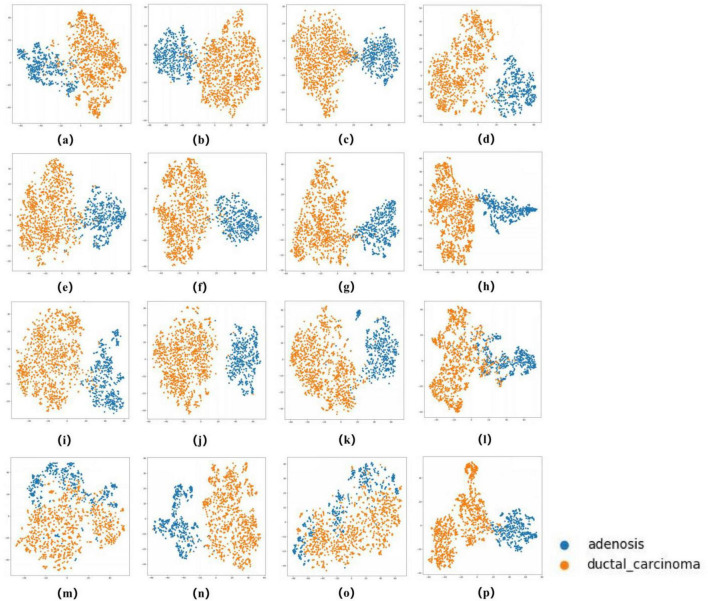
t-SNE Visualization of Embedding Features for 16 Models in a binary classification task. **(a–p)** Represent the t-SNE plots of the AlexNet, VGG16, InceptionV3, ResNet50, Densenet121, MobileNetV2, ResNeXt, RegNet, EfficientNet_B0, ConvNeXT, ViT, DINOV2,UNI (zero-shot), UNI (fine-tuned), GigaPath (zero-shot), and GigaPath (fine-tuned) models, respectively.

**FIGURE 8:**
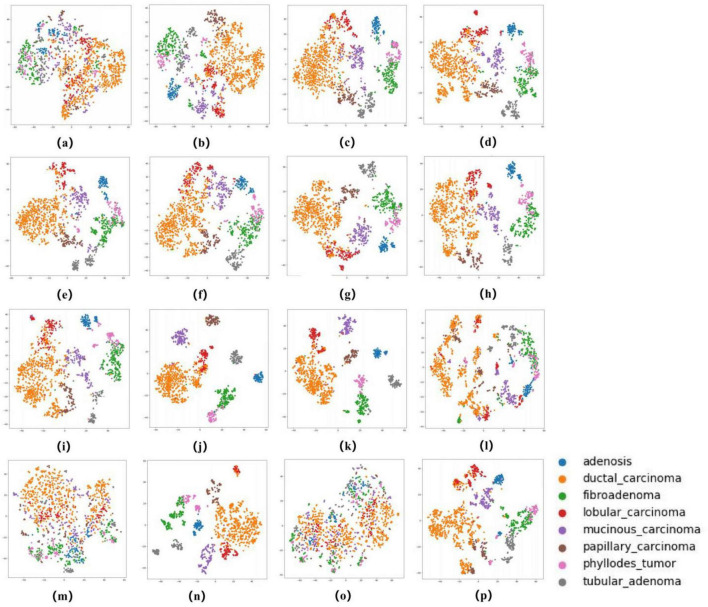
t-SNE Visualization of Embedding Features for 16 Models in an eight-class classification task. **(a–p)** Represent the t-SNE plots of the AlexNet, VGG16, InceptionV3, ResNet50, Densenet121, MobileNetV2, ResNeXt, RegNet, EfficientNet_B0, ConvNeXT, ViT, DINOV2, UNI (zero-shot), UNI (fine-tuned), GigaPath (zero-shot), and GigaPath (fine-tuned) models, respectively.

The ROC curves in Figures 5, 6 confirm ConvNeXT (36) outstanding performance in binary classification (AUC = 0.999) and the effectiveness of Transformer-based models in eight-class classification (AUC = 0.998). Feature visualization via t-SNE (Figures 7, 8) indicates that architectures following ConvNeXT achieved superior class separation compared to earlier CNN models [AlexNet (32), VGG16 (19), InceptionV3 (31)], with the eight-class clustering showing slightly reduced—though still consistent—performance trends relative to binary classification.

### 3.2 Visual interpretation of models

**FIGURE 9:**
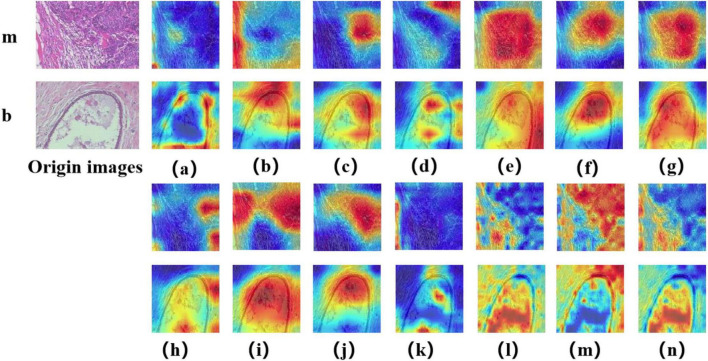
Displays Grad-CAM visualizations for 14 Models in a binary classification task. The leftmost image is the original image, with “m” representing malignant and “b” representing benign. The leftmost letter(s) represent the tumor type. **(a–n)** The heatmaps generated by the AlexNet, VGG16, InceptionV3, ResNet50, Densenet121, MobileNetV2, ResNeXt, RegNet, EfficientNet_B0, ConvNeXT, ViT, DINOV2, UNI, and GigaPath models, respectively.

**FIGURE 10:**
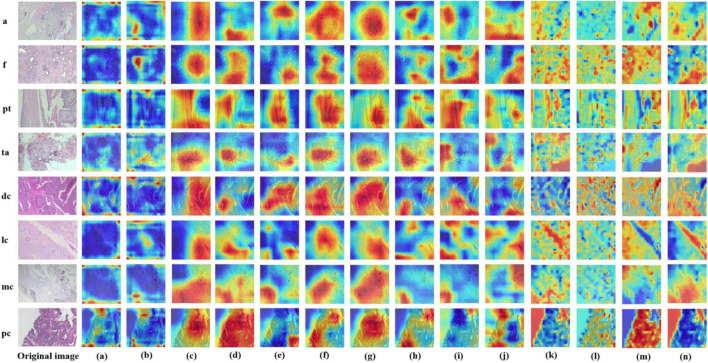
Displays Grad-CAM visualizations for 14 Models in an eight-class classification task. The leftmost image is the original image, with “m” representing malignant and “b” representing benign. The leftmost letter(s) represent the tumor type. **(a–n)** The heatmaps generated by the AlexNet, VGG16, InceptionV3, ResNet50, Densenet121, MobileNetV2, ResNeXt, RegNet, EfficientNet_B0, ConvNeXT, ViT, DINOV2, UNI, and GigaPath models, respectively.

In this study, we employed the Grad-CAM++ algorithm to generate visual heatmaps for 12 deep learning models, highlighting the most critical regions for classification decisions through weighted summation of feature mappings. As shown in Figures 9, 10, for both binary classification (benign vs. malignant) and eight-category subtype classification tasks, the top-performing models—including ResNet50 (18), RegNet (34), ConvNeXT (36) and UNI—demonstrated heatmap activations (indicated by red regions) that were precisely concentrated around pathological tissue areas. These visualization results confirm that high-performance models effectively capture diagnostically significant pathological features, with attention mechanisms showing strong alignment with clinicians’ diagnostic focus areas. Notably, ViT and its derivative models, including DINO as well as the foundation models UNI (28) and GigaPath (29) developed based on DINO, demonstrated strong feature extraction capabilities. These models effectively focused on key lesion regions while integrating broader contextual information, which may account for their superior performance in complex classification tasks. In contrast, earlier CNN models like AlexNet (32) exhibited scattered attention patterns, with some focus areas deviating from actual pathological changes—consistent with their relatively lower classification accuracy. These visual analyses provide intuitive insights into different architectures’ decision-making mechanisms while validating the clinical plausibility of high-performing models in lesion localization.

In Figures 9, 10, the models from a to p are arranged chronologically. We observe a general trend of increasing attention to prominent pathological structures over time, indicating a continuous improvement in the models’ feature extraction capabilities.

## 4 Discussion

According to the National Breast Cancer Foundation, the United States reported over 276,000 invasive and 48,000 non-invasive breast cancer cases in 2020, with projections of 287,850 new invasive cases and 51,400 DCIS cases in 2022 resulting in 43,250 deaths (42). Breast cancer is clinically categorized into four types: normal, benign (non-threatening structural changes), carcinoma in situ (localized and treatable when detected early), and invasive carcinoma (the most dangerous metastatic form) (43, 44). Current diagnostic methods include X-ray mammography, ultrasonography, CT, PET, MRI, and thermography, with pathological diagnosis through H&E-stained histopathological image analysis remaining the gold standard. Early detection is critical as 64% of cases diagnosed in initial stages demonstrate 99% survival rates, highlighting the urgent need for improved screening technologies. Two primary diagnostic approaches exist: histopathological image analysis of breast tissue morphology and genomic analysis, with the former being particularly valuable for early-stage detection.

The development of deep learning in computer vision has undergone transformative evolution from traditional CNN architectures to lightweight networks, and subsequently to Transformer-based paradigms. AlexNet (32) (2012) pioneered deep learning applications in image classification, while VGG16 (19) (2014) enhanced feature extraction capabilities through increased network depth, albeit with substantial computational demands. InceptionV3 (31) (2015) introduced multi-scale convolution to reduce computational redundancy and improve feature representation efficiency. ResNet50 (18) (2015) addressed the vanishing gradient problem in deep networks through residual connections, enabling successful training of deeper architectures. Subsequent innovations included DenseNet121 (20) (2017), which employed dense connections to enhance information flow while reducing parameter redundancy, and ResNeXt (34) (2017) that improved computational efficiency through group convolutions. The growing demand for mobile computing spurred the development of MobileNetV2 (33) (2018), utilizing depth-wise separable convolutions and linear bottleneck structures for efficient lightweight design. EfficientNet_B0 (21) (2019) implemented compound scaling strategies to optimize computational costs while maintaining accuracy, whereas RegNet (35) (2020) automated efficient CNN design through neural architecture search (NAS). The field witnessed a paradigm shift with ViT (22) (2020), which introduced Transformer architectures to visual tasks through self-attention mechanisms for global feature modeling, albeit requiring extensive training data. ConvNeXT (36) (2022) subsequently modernized CNN architectures by incorporating ViT design principles, employing large kernels and optimized activation functions to maintain competitiveness in the Transformer era. This evolutionary trajectory demonstrates continuous innovation in computer vision, progressing from depth expansion (VGG16) to computational optimization (Inception, ResNet, MobileNet), architectural innovation (DenseNet, ResNeXt), lightweight automation (EfficientNet, RegNet), Transformer disruption (ViT), and ultimately CNN modernization (ConvNeXT). The DINOv2 (23) model further advanced ViT architectures by incorporating register tokens and pretraining on 142 million images, generating powerful visual features that achieve strong performance across diverse computer vision tasks without fine-tuning.

In recent years, Transformer-based models have made remarkable progress in the medical field, expanding from natural language processing to various modalities such as medical images and time-series data, demonstrating powerful modeling capabilities and strong generalization. In medical image segmentation, MedSAM (45), a large-scale pre-trained foundation model built on over 1.5 million image-mask pairs, achieves high accuracy and robustness across diverse internal and external tasks, overcoming the limitations of task-specific designs and promoting the development of general-purpose segmentation models. In visual Transformer architectures, Swin Transformer (46) introduces a hierarchical structure with shifted windows, balancing computational efficiency and representation power, and has become a widely adopted backbone for downstream tasks such as classification, detection, and segmentation in medical imaging. For medical time-series analysis, Medformer (47) leverages multi-granularity patching and self-attention mechanisms tailored for EEG and ECG signals, significantly improving the classification of diseases such as Alzheimer’s, Parkinson’s, and myocardial infarction. In computational pathology, UNI (28) is a general-purpose pathology model pretrained on over 100,000 H&E-stained whole slide images (WSIs), enabling resolution-agnostic classification, robust subtype generalization, and few-shot learning across multiple tissue types and cancer subtypes. Meanwhile, Prov-GigaPath (29), the first foundation model for full-slide pathology, incorporates LongNet to model gigapixel-scale data from over 1.3 billion patches, achieving state-of-the-art performance on 25 out of 26 benchmarks. These advances highlight the versatility of Transformer-based models in handling high-resolution, highly structured medical data and modeling complex time-series and gigapixel pathology slides, offering strong support for precision diagnosis and personalized healthcare.

In breast cancer applications, deep learning has demonstrated significant clinical value. Wang et al. (48) developed DeepGrade, a histologic grading model using whole slide imaging (WSI) for risk stratification of NHG2 breast cancer patients, proving its independent prognostic value for clinical decision-making. Yu et al. (49) proposed a 5G+ telemedicine solution incorporating edge computing and Inception-v3 (31) transfer learning, achieving 98.19% diagnostic accuracy in remote areas. Arya et al. (50) implemented a gated attention deep learning model with random forest classifier for multimodal breast cancer prognosis prediction, demonstrating 5.1% sensitivity improvement on METABRIC and TCGA-BRCA datasets. Jaroensri et al. (51) created a deep learning system for scoring three critical histologic grading features, showing improved pathologist concordance and prognostic performance. While these applications employ various base models as encoders with architectural modifications, we contend that medical imaging applications may benefit more from advanced image preprocessing techniques to enhance input quality, which can sometimes surpass the benefits of model architecture modifications alone.

In this study, we systematically evaluated the performance of both CNN-based deep learning models and vision transformers on the BreakHisv1 dataset for breast cancer histopathological diagnosis. In the binary classification task, where the complexity of discrimination is relatively low, most models demonstrated excellent performance. Notably, CNN-based architectures such as ResNet50, RegNet, and ConvNeXT achieved particularly strong results, with ConvNeXT attaining the best overall performance—benefiting from its incorporation of design elements inspired by the Vision Transformer (ViT), which enhances global feature modeling. ConvNeXT reached an accuracy of 99.2% (95% CI: 98.3%–1), a specificity of 99.6% (95% CI: 99.1%–1), an F1-score of 99.1% (95% CI: 98.0%–1), a Cohen’s Kappa of 0.983 (95% CI: 0.960–1), and an AUC of 0.999 (95% CI: 0.999–1). These results suggest that, in relatively simple binary classification tasks, CNN-based models tend to outperform pure Transformer architectures, possibly due to their stronger inductive biases and efficiency in local feature extraction. In contrast, the eight-class classification task posed greater complexity, leading to more evident performance differentiation among models. While both CNN and Transformer-based models performed competitively, the fine-tuned Transformer-based foundation model UNI achieved the highest overall performance, with an accuracy of 95.5% (95% CI: 94.4%–96.6%), specificity of 95.6% (95% CI: 94.2%–96.9%), F1-score of 95.0% (95% CI: 93.9%–96.1%), Cohen’s Kappa of 0.939 (95% CI: 0.926–0.952), and AUC of 0.998 (95% CI: 0.997–0.999). In contrast, directly applying the encoder of foundation models such as UNI and GigaPath in a zero-shot manner, without fine-tuning, led to substantially reduced performance in both binary and multi-class settings.

In medical image classification tasks, the performance differences between CNN-based models, Transformer-based models (including foundation models) primarily stem from the alignment between task complexity and model characteristics. For binary classification tasks, the key challenge typically lies in extracting localized discriminative features (e.g., lesion boundaries or textures). The inductive biases of CNNs (translation invariance, local receptive fields) enable them to efficiently capture such spatial hierarchical features with lower computational costs. In contrast, while Transformers excel at modeling long-range dependencies, binary classification tasks often require limited global context, making their self-attention mechanisms computationally inefficient without proportional performance gains—leading to inferior results compared to CNNs. However, Transformer-based foundation models (e.g., UNI) can partially compensate for this architectural redundancy through large-scale pretraining, which provides generalized representations, and fine-tuning adaptability, allowing them to outperform CNNs in certain scenarios. For eight-class classification tasks, the decision boundaries become more complex, requiring fine-grained feature interactions. Here, Transformers’ global attention mechanisms and high-level relational modeling capabilities become advantageous: they can simultaneously integrate local and global information to resolve inter-class similarities. Foundation models further enhance generalization through pretrained knowledge transfer (e.g., cross-modal semantic correlations), whereas the locality constraints of CNNs may limit their ability to capture intricate discriminative patterns. In summary, the performance disparity fundamentally reflects a dynamic trade-off between task requirements (locality/globality, data scale) and architectural properties (CNNs’ hierarchical local inductive biases vs. Transformers’ flexible attention mechanisms). Foundation models partially unify these advantages through pretraining paradigms, but their efficacy remains modulated by downstream task complexity.

This study has several limitations. First, the analysis was conducted using a single dataset (BreaKHis) without incorporating multi-center or heterogeneous data sources. Consequently, the generalizability of our findings to broader clinical contexts is limited. Although the dataset includes 7,909 histopathological images, the actual number of patients is relatively small, which may impact the model’s real-world clinical applicability. Therefore, the reported results primarily reflect the relative performance of different model architectures on the BreaKHis dataset rather than their true diagnostic utility in breast cancer. Second, due to the vast number of deep learning models available, we did not exhaustively evaluate all existing architectures. Instead, we selected a subset of representative models from various stages of development, focusing on both CNN-based and Transformer-based approaches. As such, this study should be regarded as an initial exploration of the performance differences between model families, rather than a comprehensive assessment. Future studies could expand in several directions: (1) incorporating multi-center and multimodal pathological datasets to improve model robustness and clinical generalizability; (2) integrating additional data sources such as clinical notes and genomic profiles to explore the potential of multi-modal fusion models in breast cancer diagnosis; (3) developing more efficient, lightweight, and interpretable architectures to meet the practical demands of clinical deployment; and (4) conducting thorough evaluations of model fairness and performance consistency across different populations and pathological subtypes to ensure reliable and equitable real-world applications of AI in pathology.

## 5 Conclusion

In summary, this study systematically evaluated the performance of mainstream deep learning architectures, including both CNN-based and Transformer-based models, for breast cancer histopathological image classification. For the binary classification task (benign vs. malignant), we observed progressive performance improvement from early models like AlexNet to ConvNext, where the introduction of residual connections enabled effective training of deeper networks without gradient vanishing/explosion issues, significantly enhancing feature extraction capability through increased parameters. Post-ResNet architectures all demonstrated comparable performance, with their unique characteristics making them suitable for different application scenarios—for instance, MobileNet and EfficientNet’s compact design facilitates deployment on mobile devices with limited computational resources. The Transformer-based models (ViT and DINOv2) showed slightly inferior performance compared to CNN-based model in this task, potentially due to: (1) the task’s inherent complexity not requiring extremely large parameter counts, and (2) limited sample size hindering effective fine-tuning of these large-scale models pretrained on massive image datasets.

For the more challenging eight-class classification task, foundation model UNI achieved optimal performance with an AUC of 0.998. The increased task complexity likely contributed to the Transformer-based models matching CNN performance in this scenario, as both architecture types demonstrated equally strong classification capability. These findings suggest that while CNN architectures remain highly effective for fundamental histopathological classification tasks, Transformer-based models become competitive when handling more complex, fine-grained classification challenges. The comparable performance among modern architectures indicates that practical considerations like computational efficiency and deployment constraints may outweigh marginal accuracy differences in clinical implementation.

## Data Availability

The data that support the findings of this study are available from the corresponding author upon reasonable request.
